# A Deep-Learning-Based Artificial Intelligence System for the Pathology Diagnosis of Uterine Smooth Muscle Tumor

**DOI:** 10.3390/life13010003

**Published:** 2022-12-20

**Authors:** Haiyun Yu, Shaoze Luo, Junyu Ji, Zhiqiang Wang, Wenxue Zhi, Na Mo, Pingping Zhong, Chunyan He, Tao Wan, Yulan Jin

**Affiliations:** 1 Department of Pathology, Beijing Obstetrics and Gynecology Hospital, Capital Medical University, Beijing Maternal and Child Health Care Hospital, Beijing 100003, China; 2 School of Biomedical Science and Medical Engineering, Beihang University, Beijing 100191, China

**Keywords:** smooth muscle tumor, leiomyosarcoma, artificial intelligence, training set

## Abstract

We aimed to develop an artificial intelligence (AI) diagnosis system for uterine smooth muscle tumors (UMTs) by using deep learning. We analyzed the morphological features of UMTs on whole-slide images (233, 108, and 30 digital slides of leiomyosarcomas, leiomyomas, and smooth muscle tumors of uncertain malignant potential stained with hematoxylin and eosin, respectively). Aperio ImageScope software randomly selected ≥10 areas of the total field of view. Pathologists randomly selected a marked region in each section that was no smaller than the total area of 10 high-power fields in which necrotic, vascular, collagenous, and mitotic areas were labeled. We constructed an automatic identification algorithm for cytological atypia and necrosis by using ResNet and constructed an automatic detection algorithm for mitosis by using YOLOv5. A logical evaluation algorithm was then designed to obtain an automatic UMT diagnostic aid that can “study and synthesize” a pathologist’s experience. The precision, recall, and F1 index reached more than 0.920. The detection network could accurately detect the mitoses (0.913 precision, 0.893 recall). For the prediction ability, the AI system had a precision of 0.90. An AI-assisted system for diagnosing UMTs in routine practice scenarios is feasible and can improve the accuracy and efficiency of diagnosis.

## 1. Introduction

Uterine smooth muscle tumors (UMTs) are the most common tumors of the female genital tract, with an incidence of approximately 70% in women aged >40 years [1,2]. The *World Health Organization Classification of Tumours of Female Reproductive Organs* (fourth edition, 2014) classifies UMTs into three main categories: leiomyoma (including specific subtypes), leiomyosarcoma, and smooth muscle tumors of uncertain malignant potential (STUMP) [3]. These classifications are still used in the fifth edition published in 2020.

The diagnostic criteria for smooth muscle tumors include cytological atypia, mitoses, coagulative tumor cell necrosis, tumor border, and vascular invasion [4,5,6]. The three main criteria for diagnosis are the heterogeneity of tumor cells, mitoses, and tumor coagulative necrosis. The accurate accounting of mitoses is particularly important in differentiating leiomyosarcomas from certain subtypes of leiomyomas (e.g., leiomyomas with bizarre nuclei and mitotically active leiomyomas) or STUMPs. However, the judgment of these three main criteria is somewhat subjective for pathologists, especially mitoses, which are often interfered with by nuclear fragmentation, apoptosis, and inflammatory cells with irregular nuclei. This is poorly reproducible and time-consuming for pathologists and may ultimately result in an incorrect diagnosis.

With the development of computer technology and medical image analysis algorithms, it has become possible to use artificial intelligence (AI) to analyze whole-slide images and to perform early screening and diagnosis for tumors [7,8,9]. Several studies have verified the effectiveness of AI in the pathological diagnosis of tumors in different organs, such as lung cancer, breast cancer, prostate biopsy, and mesothelioma [10,11,12,13,14,15]. The current study aimed to analyze the morphological characteristics of digitally scanned sections of UMTs and build an AI diagnosis system for UMTs by using a computerized deep learning network model for image detection and recognition to assist pathologists in improving diagnostic accuracy and efficiency.

## 2. Materials and Methods

**Figure 1:**
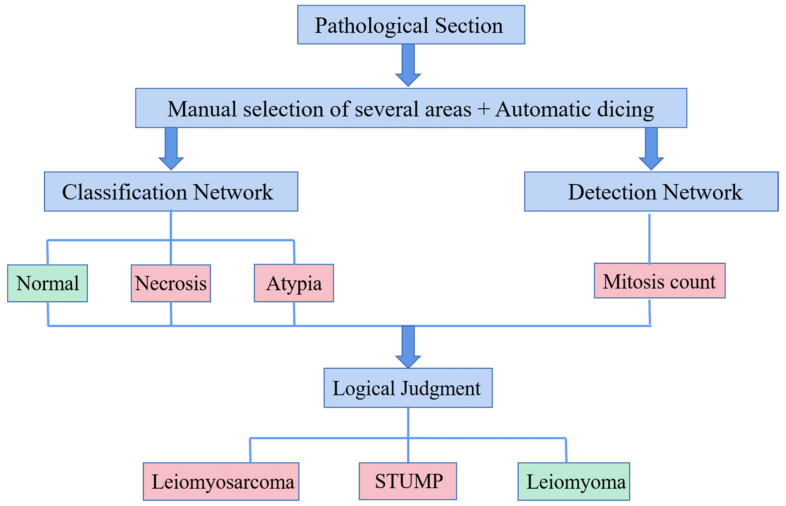
Flowchart of AI-assisted diagnosis of UMTs.

Figure 1 presents the methods of this study. After data preparation and labeling, the classification and detection models were trained to obtain an automatic UMT diagnostic aid that can “study and synthesize” a physician’s experience.

### 2.1. Data Set

The Ethics Committee of Beijing Obstetrics and Gynecology Hospital approved the study. The requirement for informed consent was waived because the reports were anonymized. Overall, 29 cases of leiomyosarcomas, 5 cases of STUMP, and 24 cases of leiomyomas (including 20 cases of conventional leiomyomas and 4 cases of lipoleiomyomas) were collected. All patients were diagnosed by the Department of Pathology of Beijing Obstetrics and Gynecology Hospital from May 2016 to May 2021. The inclusion criteria were as follows: clinical diagnosis of uterine occupancy with mass resection or total hysterectomy and pathological diagnosis of smooth muscle tumors, including leiomyomas, STUMP, and leiomyosarcomas. The exclusion criteria were as follows: patients who (1) had received preoperative radiotherapy or chemotherapy before surgery, (2) were diagnosed with uterine leiomyosarcomas exhibiting a predominant epithelioid appearance, (3) were diagnosed with myxoid leiomyosarcoma or leiomyomas with bizarre nuclei, (4) had used hormonal drugs, or (5) were pregnant.

This study used 233 digital slides of leiomyosarcomas, 108 digital slides of leiomyomas, and 30 digital slides of STUMP stained using hematoxylin and eosin (HE). Two pathologists selected and read all slides, and all data were strictly desensitized.

Aperio ImageScope software (Vista, CA, USA) was used for the annotation of digital sections. To train the deep learning model, the regions of necrotic, cytological atypia, collagen, blood vessels, and a certain range of mitosis targets in the digital sections were first annotated by pathologists. To facilitate the subsequent detection of nuclear fractures, the area of the field under the microscope was roughly converted into 10 square areas with 969-pixel borders, according to the method of counting nuclear fractures in 10 high-power fields (HPF, d = 0.55 mm). Five pathologists randomly selected a marked region in each section that was no smaller than the total area of 10 HPF in the digital sections. We labeled the areas of necrosis (N), vascular (x), collagen (j), and mitoses (h).

### 2.2. Deep Learning Models

The deep learning modern model was established on the basis of multiple convolutional neural network (CNN) feature extraction backbones and the image features of UMTs [16,17]. The images were divided into small-scale cuts that were 224 pixels × 224 pixels or 128 pixels × 128 pixels in size. An 18-layer residual network model was used to train and test the automatic classification on a server equipped with four NVIDIA Tesla v100 graphics cards to determine whether the slices had cytological atypia or necrosis. A small-scale detection network was built directly by using the YOLOv5s model with two NVIDIA RTX 3090 graphics cards to obtain a mitosis detection network. The YOLOv5s network is the network with the smallest depth and the smallest width of characteristic graph in the YOLOv5 series; therefore, this model has a faster training and prediction speed than other models and is widely used in medical case diagnosis research.

The classification and mitoses detection results were logically judged to obtain the final diagnosis. All of these were automatically handled by the deep learning model.

### 2.3. Evaluation Metrics

Our evaluation metrics included the following: (1) accuracy: (TP + TN)/(TP + FP + TN + FN); (2) precision: TP/(TP + FP); (3) recall: TP/(TP + FN), which is the proportion of correct positive predictions for all positive samples; and (4) F1 index: two multiples of the summed mean of precision and recall.


(1) $$F1\;\mathrm{Index}=\frac{2\times \mathrm{recall}\times \mathrm{precision}}{\mathrm{recall}+\mathrm{precision}}$$

TP indicates that the sample is positive and that the prediction result is also positive. FP indicates that the sample is negative, but the prediction result is wrongly interpreted as positive. TN indicates that the sample is negative and that the prediction result is also negative. FN indicates that the sample is positive, but the prediction result is wrongly judged as negative. The accuracy rate and F1 index can be used to measure the overall classification performance of the model, and a value that is closer to one indicates a better model. Accuracy, which is also known as the accuracy check rate, indicates the proportion of TPs among the positive samples detected by the model. Recall, which is also known as the full check rate, indicates the proportion of positive samples accurately detected by the model among all positive samples.

## 3. Results

### 3.1. Image Annotation

Among the patients with leiomyosarcoma, 2 had moderate cytological atypia, 5 had moderate–severe cytological atypia, and 20 had severe cytological atypia. A total of 24 patients had necrosis with an average count of more than or equal to 10 mitoses in 10 high-magnification fields (≥10/10 HPF).

**Figure 2:**
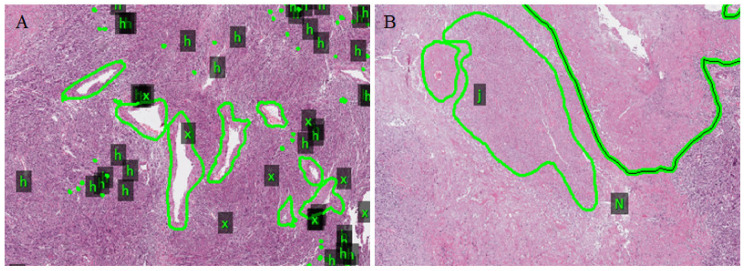
Annotations were made in tumor areas, including necrosis (N), blood vessels (x), collagen area (j), and mitotic (h). (**A**,**B**) represent different areas of the leiomyosarcoma image (Original magnification: 40×).

A total of 140 images of 19 patients with leiomyosarcoma were selected as the training set and were labeled by pathologists. The cellular regions of the tumor in the digital pathology slices were classified as normal or mild atypia, tumor necrosis, tumor cytological atypia (moderate to severe), collagen area, and vascular regions (Figure 2).

### 3.2. Automatic Classification of Necrosis and Tumor Cytological Atypia

**Figure 3:**
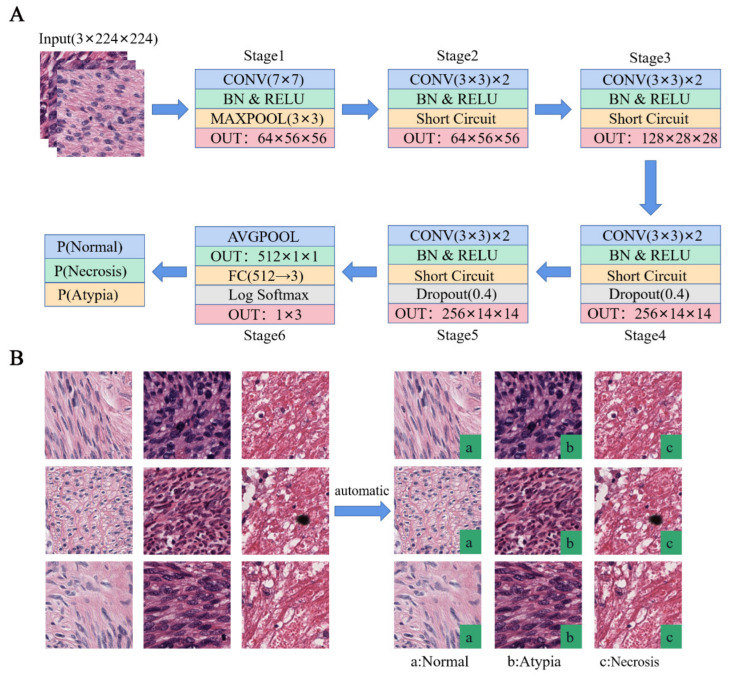
Automatic classification of necrosis and tumor cytological atypia: (**A**) classification network structure; we performed a 7 × 7 normal convolution and four 3 × 3 residual channel convolutions and used global average pooling and full connection; (**B**) results of the classification network.

By using image data that were manually labeled by physicians, a classification model was constructed using a residual network. Among the patients with leiomyosarcoma, 140 slice images of 19 patients were selected as the training set. Some 93 slice images of another 10 patients with leiomyosarcoma were selected as the test set, in which different areas were intercepted according to necrosis and nuclear anomaly annotation. Images of normal or mild cytological atypia samples were obtained from patients with leiomyoma (57 slices of 19 patients). The target regions in the slices were intercepted and cropped into small blocks of 224 pixels × 224 pixels (Figure 3). The final image blocks of the training set obtained 6418 images of moderate and severe cytological atypia, 2593 images of normal or mild atypia, and 13,266 images of necrosis. The test set contained 1200 images of each.

The test results for the model after training are shown in Table 1. The table shows that the classification network can correctly classify normal images, nuclear atypia, and necrotic images. In addition, all classification indices reached more than 0.920.

### 3.3. Automatic Detection of Mitoses

**Figure 4:**
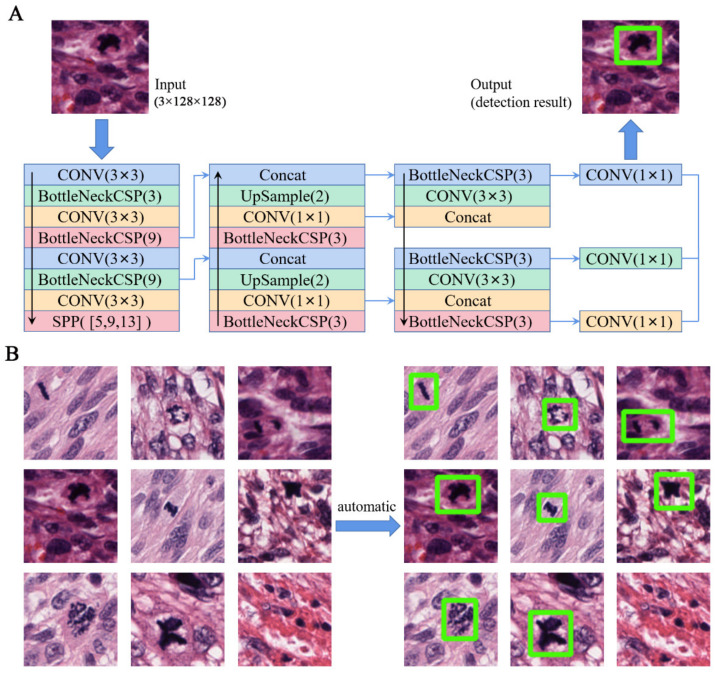
Automatic detection of mitoses: (**A**) detection network structure; we used the fifth generation YOLO algorithm as the basic framework. BottleNeck Cross Stage Partial and Spatial Pyramid Pooling were used to optimize the feature extraction structure and improve the accuracy of mitosis detection; (**B**) the results of the detection network. The green box refers to the mitosis target.

The microscopic counting of mitoses requires the high-resolution observation of pathological sections to identify various morphological targets of nuclear division at the cellular level (Figure 4). On the basis of this working idea, the YOLOv5s model in the one-stage detection mode was used for the construction and training of the detection network in this project. The hardware device was two NVIDIA RTX 3090 graphics cards.

First, the target field in the physician-labeled slice was intercepted using the program. The coordinates were then repositioned in the intercepted result, with the upper left corner of the intercepted region as the coordinate origin and the positive direction to the right and down, respectively. Second, the manually labeled position of each nuclear split was recorded and saved. Finally, we recorded the coordinates of the center point of each nuclear split region, the length of the region, and the width of the region using rectangular positioning boxes. The size of each cut block was 128 pixels × 128 pixels (Figure 4).

According to the pathologist’s annotation, 2000 blocks were used as the training set, which comprised 1500 cuts containing nuclear mitoses targets and 500 cuts with apoptotic body or other interfering factors. A total of 1000 blocks were used as the test set, which comprised 525 cuts containing mitosis targets and 475 normal cuts. In the detection model, the max-det value was set to one to ensure that, at most, one nuclear split target was detected in each cut block (128 pixels × 128 pixels). The confidence threshold was set to 0.6, and it was considered correctly detected when the intersection over union value of the detection box and manual marker box overlap was 0.55. The detection network could accurately detect the mitoses with 0.938 precision, 0.913 accuracy, 0.893 recall, and 0.915 F1 index (Table 2), which could meet the requirements in practical applications. After detecting the target, the program could further calculate the number of mitoses required to obtain the target result.

### 3.4. AI for Logical Judgment

**Figure 5:**
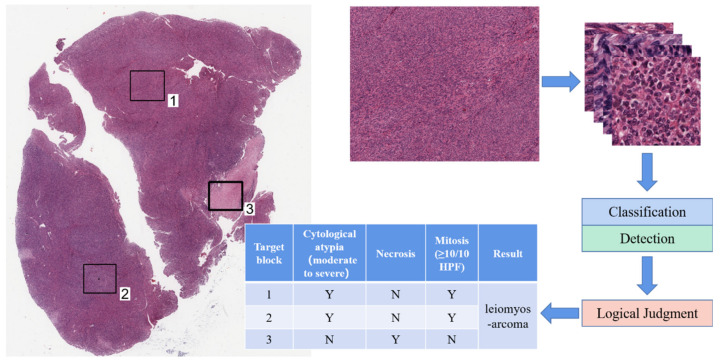
Flowchart of the automated diagnosis of UMTs by our proposed method. “Y” and “N” in the table indicate that the item is positive and negative, respectively.

To test whether the AI-aided diagnosis system can make accurate and logical judgments, we combined the automatic classification model with the automatic detection model to perform overall detection and logical judgments on pathological sections (Figure 5). We selected 10 cases of leiomyosarcoma, 5 cases of STUMP, and 5 cases of leiomyoma(1–3 sections for each case) for testing. Among them, one case of leiomyosarcoma was misdiagnosed as STUMP, and one case of STUMP was misdiagnosed as leiomyoma; the others were consistent with the pathologist’s diagnosis, with a total precision of 0.900 (Table 3). For 0.24 mm^2^ or 10 HPF of 0.55 mm in diameter, the computational times of the proposed network model for automatic classification, automatic detection, and logical judgment were 1.7, 1.5, and 0.1 s, respectively.

## 4. Discussion

The significance of using deep learning models is that automatic analysis can be obtained by learning from the samples, and the empirical knowledge of different pathologists can be synthesized. Repetitive and empirical tasks can be handed over to machines for assisted analysis. By using the computerized deep learning of digital pathological sections of UMTs, the following functions were achieved in this project: (1) automatic discriminative analysis of cytological atypia and tumorigenic necrosis of tumor cells, (2) automatic detection and counting of mitoses, and (3) logical judgment of the results obtained from the classification and detection networks to make a diagnosis.

Smooth muscle tumors are common tumors of the female reproductive system and most often occur in the uterus, followed by the cervix; broad ligament; and occasionally in the vagina, ovaries, fallopian tubes, and vulva [18]. At present, the properties of smooth muscle tumors are mainly based on the heterogeneity of tumor cells, mitosis, and tumor coagulative necrosis [3]. For example, in coagulative necrosis, a UMT with mild cytological atypia is diagnosed as a leiomyosarcoma if the mitotic count is ≥10/10 HPF; otherwise, it is diagnosed as a STUMP. In the absence of coagulative necrosis, if the tumor cells show diffuse moderate-to-severe atypia and the mitotic count is ≥10/10 HPF, the diagnosis is leiomyosarcoma. However, when only one of the conditions is met, the diagnosis is STUMP. When necrosis is lacking and cytological atypia is not obvious or only focally mild, the accuracy of the mitotic count is paramount. Tumors lacking cytological atypia and tumor cell necrosis but with ≥15 mitoses/10 HPF should be diagnosed as STUMP. Therefore, cytological atypia, mitotic count, and tumor cell necrosis play important roles in diagnosing UMTs. However, due to subjectivity, the consistency of interpretation between different pathologists is poor. In addition, counting the mitoses of tumor cells is time-consuming and labor intensive, which affects the accuracy and efficiency of diagnosis. In the classification of UMTs, STUMPs show morphological features that exceed the criteria for leiomyoma or its subtypes but are insufficient for a diagnosis of leiomyosarcoma. This issue often puzzles pathologists. However, the cytological atypia, necrosis, and mitosis in leiomyosarcoma and leiomyomas are relatively clear. We try to establish an AI judgment standard by observing the morphological characteristics of these two categories such that the diagnosis of STUMPs will be more objective.

In recent years, rapid development in the field of AI, especially deep learning (e.g., a CNN), has provided more possibilities for the establishment of intelligent computer-aided diagnostic systems based on pathological image analysis [19,20]. Deep learning is a new field in machine learning research in which higher-level attribute classes or features are formed by combining lower-level features into more abstract ones. Several studies and clinical practices are attempting to integrate AI and pathological image analysis to achieve intelligent detection and diagnosis and overcome the shortcomings of manual reading visual fatigue to improve diagnostic accuracy. Therefore, they have important clinical value and application prospects. For example, Song et al. developed an assisted diagnostic system using AI for gastric adenocarcinoma biopsy specimens. The deep CNN was trained on 2123 slides of digital pathology slices stained using hematoxylin and eosin (HE). It achieved a sensitivity of approximately 100% and an average specificity of 80.6% on a real-world test data set of 3212 slides of digital pathology [11]. In other tumors, such as esophageal cancer, lung cancer, and prostate cancer, AI based on deep learning has also achieved good detection results [21,22,23]. ResNet is a classic residual neural network and has an excellent performance in image classification tasks, including ResNet-18, ResNet-50, and other frameworks [24]. In the pre-experiments on our data set, we used ResNet-18, ResNet-34, and ResNet-50 for the classification network detection. It showed that the 18-layer residual network achieved the best classification performance. Therefore, in this experiment, an 18-layer residual network model was adopted for the model building to detect the tumor cytological atypia and tumor necrosis. The specific process is shown in Figure 3. The classification network achieved the correct classification of normal images, nuclear heterotypes, and necrotic images through testing. Furthermore, the classification performance was good, with all classification indexes reaching more than 92%. This shows that a deep-learning-based AI system can detect cytological atypia and necrosis of smooth muscle tumors.

The identification and counting of mitotic images are crucial for the differential diagnosis of benign and malignant smooth muscle tumors. Computer experts have developed several methods for mitotic detection, such as the maximized inter-class weighted mean, CNN, and YOLOv5 [25,26,27]. YOLOv5 was proposed in 2020 and is one of the latest achievements of the YOLO series of detection algorithms in the one-stage detection framework. It contains YOLOv5s, YOLOv5m, and other frameworks and has excellent detection accuracy and speed in target detection tasks. Our pre-experiment on the sub-data set showed that YOLOv5s had the best detection performance, so we chose YOLOv5s as the detection network in this experiment, as shown in Figure 4. The precision and recall steadily improved and approached one, which was used to measure the performance of the detection network during training. This indicated that the network was well trained. The detection network can detect mitoses accurately, which meets the requirements of practical applications.

We achieved automatic discrimination and classification of nuclear atypia and tumor cell necrosis through training in the classification network. The detection network makes it possible to detect and count mitoses automatically. We combined the automatic classification and automatic detection models and performed overall detection and logical judgment on some pathological sections, shown in Figure 5. In the detection experiments, 20 cases of UMTs, including 10 cases of leiomyosarcomas, 5 cases of STUMPs, and 5 cases of leiomyoma, were tested. Except for one leiomyosarcoma misdiagnosed as STUMP, the results were consistent with the pathologist’s diagnosis, with 0.90 precision. Our study initially explored the feasibility of using histopathological AI-assisted systems to diagnose UMTs, which can assist pathologists in making judgments and improve the efficiency and accuracy of diagnosis.

This study had some limitations. First, the number of images in the categories is small. To improve the performance of our diagnostic system, a larger data set is required. Second, our experiments were conducted mainly on UMTs, and the morphological features of other spindle cell tumors in the uterus, such as endometrial mesenchymal sarcoma, inflammatory myofibroblastoma tumor, and perivascular cell tumor, were not learned and judged by AI. Therefore, it is still necessary to manually select the slides of pathological images to be analyzed. Owing to the limited number of cases, the computer learning and judgment of cell-rich leiomyomas myxoid leiomyosarcoma, leiomyomas with bizarre nuclei, and epithelioid leiomyosarcoma are not yet convincing. Finally, the false detection rate of the model for detecting mitoses was relatively high. Given that clinical information, immunohistochemical markers, and molecular detection results can help affect pathological diagnoses [28,29], especially for relatively difficult cases, it is necessary to combine this information with the AI system. This is a difficult issue in automatic histopathological diagnosis. In subsequent experiments, we should continue to expand the sample size and combine immunohistochemistry and molecular results with AI to improve the diagnostic accuracy and automation of the model.

## 5. Conclusions

The criteria for UMT diagnosis are very complex. Furthermore, UMT diagnosis is time-consuming and error prone. This study proposes an AI-aided diagnosis and evaluation system for UMTs based on deep learning. The algorithms for the automatic classification of necrosis and tumor cytological atypia and the automatic detection of mitoses were proved to be effective. By analyzing whole-slide images, an AI system can judge the properties of UMTs and make logical conclusions. This system may provide a new, comprehensive, and intelligent method for pathological diagnosis and may generate new ideas for advancing interdisciplinary collaborative research on clinical medical problems. However, there are still areas that can be improved, and our current work is mainly based on the pathological image slices of a certain size. Therefore, it is necessary to manually frame a certain area to be analyzed on a slice. Achieving automatic application at the WSI level requires further research. In addition, studies should be conducted on the improvement of the depth learning algorithm to further improve the accuracy of the system and ensure its clinical practicality.

## Figures and Tables

**Table 1:** The accuracy, precision, recall, and F1 score of various categories in the test set.

Types	Accuracy	Precision	Recall	F1 Index
Moderate-to-severe cytological atypia vs. normal and mild cytological atypia	0.962	0.928	0.998	0.963
Tumor necrosis vs. normal	0.947	0.930	0.969	0.949

**Table 2:** The precision, recall, accuracy, and F1 index of mitoses detection in the test set.

	Detected as Mitosis	Detected as Normal or Apoptotic Body	Total	Precision	Recall	Accuracy	F1 Index
Actually mitotic	469	56	525	0.938	0.893	0.913	0.915
Actually normal or apoptotic body	31	444	475				
Total	500	500	1000				

**Table 3:** The accuracy, precision, recall, and F1 score of various categories in the test set.

Types	Accuracy	Precision	Recall	F1 Index
Leiomyosarcoma vs. STUMP and leiomyoma	0.950	1.000	0.900	0.947
STUMP vs. leiomyosarcoma and leiomyoma	0.900	0.800	0.800	0.800
Total for three categories	/	0.900	/	/

## Data Availability

The data presented in this study are available upon request from the corresponding author.
